# Preface

**Published:** 1820-12-01

**Authors:** 


### Vol. I. No. 3. DECEMBER, 1820.

### TO SUBSCRIBERS.

THE Editor begs to solicit the attention of Subscribers to the following subject:—

The immense issue of medical works from the press of this country, (as may be seen by the list of books transmitted for review), together with the journals and other works, which are now regularly received from Europe and America, by the Editor, render it absolutely impossible for him to have justice done to the current of medical literature, without the occasional indulgence of a *supplement*, consisting of two or three sheets, to be charged at the rate of *sixpence for every 16 pages*, the common ratio of the monthly journals.

With this latitude, the Editor is confident that he shall be able to render an essential benefit to his brethren in this country, the colonies, and the public services, who cannot have access to the various works and journals, now regularly transmitted from abroad to this Review.

The Editor submits this Number of the Journal for the special examination of Subscribers, under the conviction that, on a candid review of its contents, they will acknowledge the additional 1s. 6d. to be amply compensated for in additional and valuable matter. He pledges himself, that the supplemental review shall never exceed, in price, however it may in pages, the limits here drawn, and that it shall always be dispensed with, whenever the original boundary of the Journal can be made to comprehend a quantum of analyses proportioned to the works received:—the object being rather to fix and record those portions of the passing stream of science which will best bear reiterated perusal and study, than to spread before the reader a great variety of entertainment.

As for the execution of the Journal, the Editor can safely assure the public, that he has *unlimited assistance*, at all times, from a select circle of friends, with whose talents, probity, and experience, he is personally acquainted. No pains shall be spared, on his part, to embody their united exertions in the collection and diffusion of useful knowledge, and indeed, the present Number may bear witness to the

*variety of talent* which has been efficiently combined in its construction. This feature will be still more conspicuous in every future Number.

The Editor has the pleasure of informing Subscribers and Authors, that the eclectic, or leading articles, the Supplemental Reviews, and all the principal Analyses of this Journal are now regularly republished in America; while extracts from many of them are diffused through the Continental Periodicals. It must surely be gratifying to Authors to know that *full accounts* of their productions are now read, not only wherever this Journal travels, but that, by branching into new channels, in various countries, they continue to be circulated to an almost inconceivable extent. In reciprocity of intellectual commerce, this work will endeavour to collect into a focus, the prominent rays of science and useful knowledge, that beam from other and distant regions, thus alternately diffusing light by reflection, and receiving it in return.

"Un immense reseau," says an eloquent foreign medical writer, "de communication entre tous les savans de l'Europe, et meme du nouveau-monde, entretient l'eveil des esprits, *repand les decouvertes comme l'éclair qui brille soudain à tous les yeux*, *etablit cette harmonie de sensibilité moral entre toutes les amis éclairées*, et les fait participer en meme temps aux pensées les unes des autres."

Let any one contemplate the list of works (more than *thirty* in number) which have been received since last publication day, and say whether it is possible to give any satisfactory analysis of them, within narrower limits than those here assigned. And as the public seems to have sanctioned the *object* of an Analytical Review, so it is confidently hoped, that Subscribers will cherish the exertions of the conductors, by sanctioning that scope assumed in the present Number of the Journal, and without the *occasional* aid of which, their energies must be cramped, and their means of proving useful, considerably abridged. The determination of this question, however, shall be submitted to a generous and discerning public, and they will cheerfully abide by their decision.

☞ It is expected that a plate will, in general, accompany each No. of this Journal, an addition not usual in Reviews.

